# Phylogeny-based identification of *Mycoplasma genitalium* in a Nigerian population of apparently healthy sexually active female students

**DOI:** 10.11604/pamj.2022.41.71.19631

**Published:** 2022-01-25

**Authors:** Chinyere Charity Ezeanya-Bakpa, Nneka Regina Agbakoba, Ifeoma Bessie Enweani-Nwokelo, Charlotte Blanche Oguejiofor

**Affiliations:** 1 Department of Microbiology and Biotechnology, Caleb University, Lagos, Nigeria,; 2 Department of Medical Laboratory Science, Nnamdi Azikiwe University, Awka, Nigeria,; 3 Department of Obstetrics and Gynecology, Nnamdi Azikiwe University Nnewi Campus, Nnewi, Nigeria

**Keywords:** *Mycoplasma genitalium*, female students, *16SrRNA*, phylogenetic analysis, Nigeria

## Abstract

**Introduction:**

Mycoplasma genitalium is an emerging sexually transmitted pathogen. Sexually transmitted infection (STI) is still a burden for Nigerian women because it is asymptomatic. The lack of detection of M. genitalium in apparently healthy sexually active females in Nigeria is due to non-application of high throughput molecular approach. We conducted a study to identify M. genitalium in apparently healthy Nigerian female students using a phylogenetic approach.

**Methods:**

one hundred endocervical swab specimens were collected from a student population of sexually active females aged 15 - 39 years. The 16SrRNA gene V1-V3 region of M. genitalium were amplified directly from the specimens before sequencing. Maximum Likelihood (ML) phylogenetic analysis was carried out to determine genetic relatedness.

**Results:**

the prevalence of M. genitalium infection was 1% among students. The majority (59%) of them were between 20 - 24 years, with a mean age of 26.2 ± 2.66 years. High number of sexual partners, previous STI and irregular condom use were significantly associated (P < 0.05) with the prevalence of M. genitalium infection. There was extensive lack of knowledge (0%) about M. genitalium infection among the students. Phylogenetic analysis revealed three clades with different relatedness. Our study highlighted that 16SrRNA gene was a detector of M. genitalium, but it shared no phylogenetic relationship with other examined species from around the world.

**Conclusion:**

despite a low prevalence of M. genitalium infection among the understudied group, its cause was not established; consequently, prevention and control measures should be based on health education in the general population in order to limit the spread of this pathogen. To our knowledge, this is the first study to identify M. genitalium among the general population of Nigeria using a phylogenetic approach.

## Introduction

*Mycoplasma genitalium* an emerging sexually transmitted (ST) pathogen was originally isolated in 1981 [1]. Members of the genera *Mycoplasma* are characterized as small, cell wall-less bacteria exhibiting parasitism relationship with vertebrate hosts in an obligate manner. *Mycoplasma genitalium* is constituted in the vagina, cervix and endometrium of females. It is an emerging cause of sexually transmitted infections (STIs) and has been associated with urogenital infections among men and women globally [2].

The World Health Organization reports the development of nearly 448 million new cases of STIs around the globe annually [3]. However, with early diagnosis such infections are treated certainly with slight morbidity and diminished economic burden. The demonstration of the role of *M. genitalium* in male urogenital disease proved to be a significant development in the study of STIs but its role in the female reproductive tract disease remains unclear [4].

*Mycoplasma genitalium* is a serious public health concern in reproductive age women. In view that its infection is often asymptomatic in females and with minimal testing rates, the available data is likely to underrate the circulation of *M. genitalium* in the general population. *Mycoplasma genitalium*, if left undetected could result in a wide-range of complications such as chronic pelvic pain [5], ectopic pregnancy, infertility [6], increased risk for HIV transmission and have been associated as co-factors in diseases [7].

As an emerging ST pathogen generating clinically significant infections in females, few genomes have been sequenced globally till date owing to the fastidious nature of *M. genitalium* also rare population-level data on prevalence exist [8]. Thus, there is dearth of phylogenetic data to provide insights into the genetic diversity of this specie within the student population in Nigeria. Regardless of their significance in sexual health, there are only four *16SrRNA* sequenced *M. genitalium* to date. They include: the sequence of 1490 bases of the *16SrRNA* gene for *M. genitalium* G37 (type strain) and three *16SrRNA* sequenced genome of *M. genitalium* clinical isolates from Denmark, Japan and Iran [9].

Among the limited studies, most studies on *M. genitalium* have been conducted in specialized populations (usually clinics). Though these study groups are ideal for studying hypothetically new ST pathogens, there is a possibility of underestimate of the prevalence in the general population. The objective of this study was to characterize and describe the phylogenetic features of *M. genitalium* from apparently healthy sexually active female students in Edo State, Nigeria, from March to August 2018.

## Methods

**Study sites and design:** the study was a population-based, cross-sectional study where endo-cervical swab specimens were collected from apparently healthy females of reproductive age (15-39 years) for the study of *M. genitalium* using *16SrRNA* gene sequencing methods. The population comprised of undergraduate female students of the Edo State School of Health Technology, Benin city. This institution is a government-owned Tertiary Institution.

***Study population, eligibility and data collection:*** informed consent; a pre-requisite for the study was obtained from the participants. Consequently, a total of 100 consenting sexually active female students aged 15 - 39 years in a large tertiary institution in Benin City, Southern Nigeria from March 2018 to August 2018 were randomly chosen and integrated in this study. The World Health Organization (WHO) guideline on survey of *Mycoplasma genitalium* infection was not available at the time of this study; however, the guideline for sexually transmitted infection recommends that the minimum acceptable sample size for determining prevalence relies on the particular etiologies and the estimated prevalence of the pathogen(s). A sample size of 50 - 100 specimens from a given population at risk provide adequate information for useful analyses [10]. Sample size was determined at 95% confidence interval using 0.05 degree accuracy with a prevalence of 8.5% reported among females aged 15 - 45 years in South Africa [11]. Subjects menstruating, reporting previous diagnosis with human immunodeficiency virus (HIV) infection and antibiotic use within 90 days prior to the time of the study were excluded. A structured-specific questionnaire was designed to obtain data on participants´ demographic information and sexual activities.

**Sample collection and laboratory analysis:** Endo-cervical swab (ECS) specimen was collected from each subject targeted for *M. genitalium* detection. Endo-cervical swabs were sluiced in 0.4 ml of sterile Phosphate Buffered Saline (PBS; Oxoid, United Kingdom) for 30 seconds and then transported on ice at 40C. The supernatant was used for DNA extraction afterwards with commercially available extraction reagent kit QiaQuick DNA kit (Zymo Research, Germany) with compliant to the manufacturer's instructions. The commercial master mix with standard buffer OneTaq Quick load 2X (New England Bio Labs, USA) was used for the Polymerase chain reaction (PCR) amplification. High quality, desalted primer pair for *16SrRNA* gene variable region (V1 - V3) of *M. genitalium* (GPO and MGSO) were provided by Inqaba Biotechnology, Pretoria, South Africa according to previously reported design [12]. Capillary Sanger/dideoxy was performed for the sequencing of the PCR products. Genetic and phylogenetic analysis was performed with MEGA software (MEGA 7.0 version), whereas sequences were aligned using the CLUSTAL W algorithm [13]. Genetic distances between the sequences were calculated using the Tamura-Nei model [14]. Species definition was accomplished through the phylogenetic analysis of the sequenced samples and *M. genitalium* reference sequences with other previously submitted sequences on GenBank from other parts of the world.

**Statistical analysis:** the software used for data analysis of results obtained was Statistical Package for Social Sciences (SPSS) program - version 20. Descriptive simple statistical tool was used to estimate prevalence from the data obtained. The Pearson Chi-square test was used to determine the association between demographic data, sexual activities and outcome of specimen analysis; however, when expected numbers in the association table were small, Fisher´s exact test was used. Significant association was considered where probability value (p-value) was less than 0.05 (P < 0.05). Bootstrap confidence value ≥ 70% was considered significant using Hillis and Bull method [15] and aided the establishment of the genetic relatedness among isolates of *M. genitalium*. Bootstrapping analysis was done in 1,000 replicates to address bias.

**Ethical considerations:** the study was assessed and granted ethical approval by the Edo State Hospitals´ Management Board Research Ethics Committee prior to the commencement of the study. Confidentiality was conserved by the exclusion of subjects´ names.

## Results

**General characteristics of the study population:** a total of 100 sexually active female students were screened for *M. genitalium* during the study period. Detailed characterization of the study population revealed that the majority (59%) were between 20 - 24 years, followed by 17% that were between 25 - 29 years, with mean age 26.2 ± 2.66 years (Table 1). There were 64%, 58% and 73% of the participants not married, lived outside campus and had no children respectively. All the endo-cervical swab (ECS) specimens were analyzed for *M. genitalium* molecularly.

**Table 1:** general characteristics of the study population

Characteristics	Studied population n (%)
**Age (mean ± SD)**	24.4 ± 2.66
Age (years)	
15-19	7(7)
20-24	59(59)
25-29	17(17)
30-34	12(12)
35-39	5(5)
**Marital status**	
Married	12(12)
Single	88(88)
**Housing**	
Live on campus	33(33)
Live outside campus	58(58)
Live with parent/guardian	9(9)
**Children**	
Yes	32(32)
No	68(68)
**%- Percentage**	

***Mycoplasma genitalium* isolates and sequencing:** *Mycoplasma genitalium* was isolated via subjection to direct molecular analysis; the sequence and phylogenetic analysis of the *16SrRNA* of *M. genitalium* detected within the study period was sequenced. The raw sequence was subjected to editing before generating a consensus sequence, which was submitted to the National Center for Biotechnology Information (NCBI). The accession number of the *M. genitalium* was MG238565.

**Prevalence of infection and identified risk factors:** a total of 1 (1%) of the 100 female students examined had *M. genitalium* in their cervix, as shown in Table 2. The prevalence was seen among females aged 20 - 24 years but was not significantly associated (P = 0.487); likewise, other socio-demographic characteristics such as those married, had no children and lived outside campus had prevalence of *M. genitalium* although not significantly associated with P-value of 0.389, 0.678 and 0.412 respectively.

**Table 2:** association of socio-demographic characteristics, sexual activities and isolated microbe

Characteristics value	Total (n = 100)	M. genitalium infection status n (%)		P-value
		Positive	Negative	
**Age (years)**				0.487
15-19	7	0(0.0)	7(100.0)	
20-24	59	1(1.7)	58(98.3)	
25-29	17	0(0.0)	17(100.0)	
30-34	12	0(0.0)	12(100.0)	
35-39	5	0(0.0)	5(100.)	
**Marital Status**				0.389
Married	12	1(8.3)	11(91.7)	
Single	88	0(0.0)	88(100.0)	
**Housing**				0.412
Live on campus	33	0(0.0)	33(100.0)	
Live outside campus	58	1(1.7)	57(98.3)	
Live with parent/guardian	9	0(0.0)	9(100.0)	
**Children**				0.678
Yes	32	0(0.0)	32(100.0)	
No	68	1(1.1)	67(98.5)	
**Sexual activities**				0.201
Age at first intercourse ≥18	63	1(1.6)	62(98.4)	
17	14	0(0.0)	14(100.0)	
16	6	0(0.0)	6(100.0)	
≤15	17	0(0.0)	17(100.0)	
**No. of sexual partners since FI**				0.004*
≥ 4	55	1(1.8)	54(98.2)	
3	26	0(0.0)	26(100.0)	
2	10	0(0.0)	10(100.0)	
1	9	0(0.0)	9(100.0)	
0	0	0(0.0)	0(0.0)	
**Has had previous STI**				0.020*
Yes	68	1(1.5)	67(98.5)	
No	32	0(0.0)	32(100.0)	
**Has had partner with previous STI**				0.292
Yes	29	1(3.4)	28(96.6)	
No	71	0(0.0)	71(100.0)	
**Has had partner outside the country**				0.242
Yes	16	0(0.0)	16(100.0)	
No	84	1(1.2)	83(98.8)	
**Regular condom use**				0.008
Yes	19	0(0.0)	19(100.0)	
No	81	1(1.2)	80(98.8)	

No.- Number; %- Percentage; STI- Sexually transmitted infection; FI- First intercourse *P< 0.05

Data from the sexual activities of the participants revealed the prevalence of 1% for *M. genitalium* among females who had high number (≥ 4) of sexual partners since first intercourse, previous sexually transmitted infection and irregular use of condom. Of these variables, significant association (P < 0.05) was also found. On further examination, 80% of the females with history of previous STI claimed to have had symptoms like: vaginal itching, watery and smelling vaginal discharge, chronic pelvic pain, burning urination, pain during sex and abnormal vaginal bleeding; however, 86% reported self-resolving, while 15% reported the use of drugs as curative measures without a doctor´s prescription. The prevalence of *M. genitalium* was also observed among other sexual activities such as: age at first intercourse, partner with previous STI and females who have had partner(s) outside the country; though, not significantly associated with P-value of 0.201, 0.292 and 0.242 respectively.

None of the females interviewed (0%) reported knowing about *M. genitalium* infection; although reported to have little knowledge (26%) of other STIs. Five of the females (5%) indicated to being informed that asymptomatic state of infection exist among females and either abstinence or regular condom use are means of preventing STIs.

**Figure 1:**
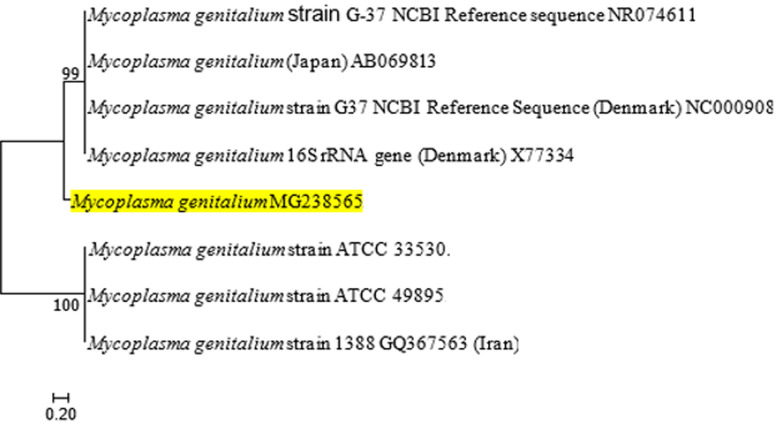
molecular phylogenetic analysis by maximum likelihood method

**The *Mycoplasma genitalium* phylogenetic framework:** to construct a partial genome *16SrRNA*-based phylogeny with *16SrRNA* sequence from this study, sequence reads were aligned to the following *M. genitalium* strains: 1 strain (NC000908) of published whole genome sequence in 1995 [16] and 1 strain (NR074611) of *16SrRNA* gene partial sequence in 2006 [17]. Both were NCBI reference sequences (*M. genitalium* G37). Five other published genomes were also included. They are: 1 isolate (X77334) from Denmark retrieved from *M. genitalium* G37 type strain in 2003 using modified Hayflick's medium [9]. One strain (AB069813) originated from urine samples among symptomatic male Japanese urethritis patients collected within 1999 and 2000 [18] whereas one strain (Strain 1388, GQ367563) was from genital swabs among 83 *Mycoplasma*-positive Iranian female patients with genital infection collected between 2007 and 2008 [19]. And finally two additional ATCC reference strains´ *16SrRNA* gene, partial sequence were also included: ATCC 33530 and ATCC 49895 [20]. The percentage of the genome covered by reads for the isolated sequenced in this study was 99.5%. Phylogenetic analysis of *16SrRNA* sequences of the isolated *M. genitalium* from this work highlighted in yellow with the 7 reference strains of *M. genitalium* obtained from GenBank showed that the *M. genitalium* isolate sequence (MG238565) from the students were not found to have significant phylogenetic relationship with the reference strains (Figure 1).

## Discussion

This study on *M. genitalium* among sexually active female students aged 15-39 years in a tertiary institution, Benin City was done using polymerase chain reaction (PCR) amplification assay, *16SrRNA* sequencing and phylogeny-based methods. The phylogeny-based assay for *M. genitalium* provide a potent tool for the foundation of etiological study of this pathogen. Genome amplification via PCR, proceeding nucleotide sequence determination and phylogenetic analysis has developed into a global technique for the identification, classification and characterization of etiological agents [18].

This study revealed 1% prevalence of *M. genitalium* among apparently healthy female students in a large tertiary institution. However, very few studies on the prevalence of *M. genitalium* in the tertiary student population have been reported outside Nigeria and neither have there been from Nigeria. This study correlates with a study in Northern Norway by Jensen *et al*. [21] in 2013, who reported 1% prevalence among 206 female college students. Furthermore, Oakeshott *et al*. [22] in 2010 reported 3.3% prevalence among 2378 female students from tertiary institutions in London. The type of specimen (self-taken vaginal swab), age of the subjects (15-27 years), number of study area (20) and the large sample size could justify the discrepancy as compared to our study. In 2008, Hamasuna *et al*. [23] reported prevalence of 2.1% among female students from three vocational schools in Miyazaki prefecture, Japan. This study does not correlate with the result from our study considering the type of the specimen (first voided urine) and the sample size (298) used as compared to our study; where *16SrRNA* gene was examined.

The risk factors for *M. genitalium* infection which promoted the acquisition of *M. genitalium* as shown in Table 2 included: High number of sexual partners, inconsistent condom use and previous STI [22]. These risk factors identified in this study were consistent with those obtained from other previously reported population-based studies; such as those reported among tertiary female students in Japan using first voided urine [23] and from a Norway based study among tertiary students [21]. Asymptomatic infection state continues to pose as a serious epidemiological challenge [24]. This is suggestive that not all infected individuals are symptomatic [25]; thereby supporting a hypothesis that *M. genitalium* infected females can spread the infection and increase the prevalence in the population due to asymptomatic manifestation of the infection. In addition to the behavioral risk factors, the transmission dynamics may contribute to the acquisition of the infection.

At the phylogenetic level, genetic relatedness was established with bootstrap confidence value ≥ 70% as statistically significant [15]. This study shows the first phylogenetic framework for the species *M. genitalium*, isolated from Nigerian students, including isolates from two continents which were reported from 1995 to 2008. The phylogenetic tree separated the understudied species into three main clades, of which Clade A comprised of the 2 genomes sequenced strains of G-37 [16, 17]. An association of the isolates metadata to the phylogeny revealed no relationship between phylogenetic position and country of origin, in all clades containing isolates from two continents studied: Europe and Asia (Japan and Iran).

Phylogenetic analysis aids in tracing the source of infection. *Mycoplasma genitalium* was found not to share phylogenetic relationship with *M. genitalium* from other parts of the world. A statistically significant relationship was found among *M. genitalium* genomes in Clade A and C. Whilst, Clade B consisting of isolated *M. genitalium* from the students in this study had no phylogenetic linkage with other understudied genomes. This implies that this specie circulating in the Nigerian environment is relatively unique. Identification and classification of *M. genitalium* phylogenetically aids in understanding its distribution and epidemiology. The strength of this study lies in the use of high-throughput *16SrRNA* gene sequencing method, which is ideal for fastidious organism. However, the study was a single-centered with limited number of participants.

## Conclusion

The prevalence of *M. genitalium* among the students in this study although relatively low had unique circulating specie. Data on phylogenetic analysis of *M. genitalium* from Nigeria can improve the understanding of their genetic diversity, likewise the source of infection. *Mycoplasma genitalium* is receiving increased attention in recent times and needs to be monitored in order to check its prevalence and source of infection in the general population. This will aid better infection control at both national and global level.

### What is known about this topic

- *Mycoplasma genitalium is a sexually transmitted pathogen of significant public health importance;*
- *Asymptomatic state in females is associated with M. genitalium infection*.

### What this study adds

- *The phylogenetic analysis of Mycoplasma genitalium from apparently healthy female students in Nigeria;*
- *One unique circulating specie have been identified;*
- *New data to the existing data on Mycoplasma genitalium infection among females in the student population have emerged*.

## References

[1] Tully JG, Taylor-Robinson D, Cole RM, Rose DL (1981). A newly discovered *Mycoplasma* in the human urogenital tract. Lancet.

[2] McGowin CL, Anderson-Smits C (2011). *Mycoplasma genitalium* an emerging cause of sexually transmitted disease in women. PLoS Pathog.

[3] World Health Organization (WHO) Sexually transmitted infections (STIs).

[4] Lis R, Rowhani-Rahbar A, Manhart LE (2015). *Mycoplasma genitalium* infection and female reproductive tract disease: a meta-analysis. Clin Infect Dis.

[5] Simms I, Eastick K, Mallinson H (2003). Association between *Mycoplasma genitalium* Chlamydia trachomatis and Pelvic Inflammatory Disease. Sex Transm Infect.

[6] Ezeanya-Bakpa CC, Agbakoba NR, Oguejiofor C, Enweani-Nwokelo IB (2021). Sequence analysis reveals asymptomatic infection with *Mycoplasma* hominis and Ureaplasma urealyticum possibly leads to infertility in females: A cross-sectional study. Int J Reprod Biomed.

[7] Adebamowo SN, Ma B, Zella D, Famooto A, Ravel J, Adebamowo C (2017). ACCME Research Group *Mycoplasma* hominis and *Mycoplasma* genitalium in the vaginal microbiota and persistent high-risk human papillomavirus infection. Front Public Health.

[8] Ezeanya CC, Agbakoba NR, Enweani IB, Oguejiofor C (2019). Predominance of cervicitis agents with minimal testing rate within the student population in Benin City, Nigeria. J Obstet Gynaecol.

[9] Jensen JS, Borre MB, Dohn B (2003). Detection of *Mycoplasma genitalium* by PCR Amplification of the 16S rRNA Gene. J Clin Microbiol.

[10] WHO (1999). Guidelines for sexually transmitted infections surveillance: Communicable disease surveillance and response.

[11] Hay B, Dubbink JH, Ouburg S, Roy C, Pereyre S, van der Eem L (2015). Prevalence and Macrolide Resistance of *Mycoplasma genitalium* in South African Women. Sex Transm Dis.

[12] Metwally MA, Yassin AS, Essan TM, Hamouda HM, Amin MA (2014). Detection, characterization and molecular typing of Human *Mycoplasma spp* from major hospitals in Cairo, Egypt. ScientificWorldJournal.

[13] Kumar S, Stecher G, Tamura K (2016). MEGA 7: Molecular Evolutionary Genetics Analysis version 7.0 for bigger datasets. Mol Biol E.

[14] Tamura K, Nei M (1993). Estimation of the number of nucleotide substitutions in the control region of mitochondrial DNA in humans and chimpanzees. Mol Biol Evol.

[15] Hillis D, Bull J (1993). An empirical test of bootstrap as a method for assessing confidence in phylogenetic analysis. Sys Bio.

[16] Fraser CM, Gocayne JD, White O, Adams MD, Clayton RA, Fleischmann RD (1995). The minimal gene complement of *Mycoplasma genitalium*. Science.

[17] Glass JI, Assad-Garcia N, Alperovich N, Yooseph S, Lewis MR, Maruf M (2006). Essential genes of a minimal bacterium. Proc Natl Acad Sci U S A.

[18] Yoshida T, Maeda S, Deguchi T, Miyazawa T, Ishiko H (2003). Rapid Detection of *Mycoplasma genitalium, Mycoplasma hominis, Ureaplasma parvum* and *Ureaplasma urealyticum* organisms in genitourinary samples by PCR-Microtiter Plate Hybridization Assay. J Clin Microbiol.

[19] Amirmozafari N, Mirnejad R, Kazemi B, Sariri E, Bojari MR, Darkahi FD (2009). Comparison of polymerase chain reaction and culture for detection of genital *Mycoplasma* in clinical samples from patients with genital infections. Saudi Med J.

[20] Volokhov DV, George J, Liu SX, Ikonomi P, Anderson C, Chizhikov V (2006). Sequencing of the intergenic 16S-23S rRNA spacer (ITS) region of Mollicutes species and their identification using microarray-based assay and DNA sequencing. Appl Microbiol Biotechnol.

[21] Jensen AJ, Kleveland CR, Moghaddam A, Haaheim H, Hjelmevoll S, Skogen V (2013). Chlamydia trachomatis, *Mycoplasma genitalium* and Ureaplasma urealyticum among students in northern Norway. J Eur Acad Dermatol Venereol.

[22] Oakeshott P, Aghaizu A, Hay P, Reid F, Kerry S, Antherton SI (2010). Is *Mycoplasma genitalium* in women the New Chlamydia? A community-Based prospective cohort study. Clin Infect Dis.

[23] Hamasuna R, Imai H, Tsukino H, Jensen JS, Osada Y (2008). Prevalence of *Mycoplasma genitalium* among female students in vocational schools in Japan. Sex Transm Infect.

[24] Wiesenfeld CH, Manhart LE (2017). *Mycoplasma genitalium* in Women: Current Knowledge and Research Priorities for this recently emerged pathogen. J Infect Dis.

[25] Smieszek T, White PJ (2016). Apparently-different clearance rates from cohort studies of *Mycoplasma genitalium* are consistent after accounting for incidence of infection, recurrent infection and study design. PLoS One.

